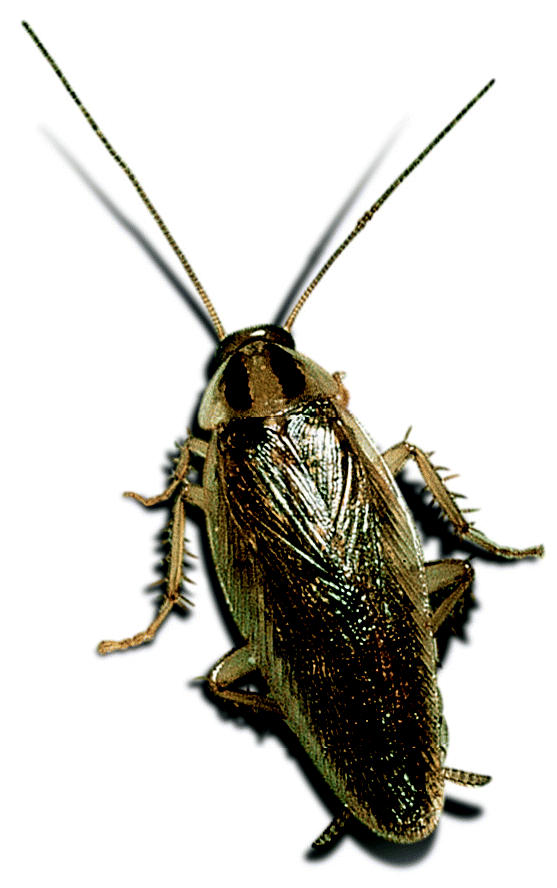# Legislation: NYC Adopts Pesticide Laws

**Published:** 2005-10

**Authors:** Luz Claudio

In response to the growing evidence that chemical pesticide use has potential human health consequences, New York City has adopted two new laws that aim to reduce exposures to toxic pesticides. The pesticide phase-out under these laws, signed in May 2005, will be complete by November 2006.

Under the NYC Pesticide Reduction Law, city agencies and their contractors must phase out the use on city property of pesticides that are known or suspected to cause cancer or developmental effects, and must adopt less toxic alternatives for pest control. Under the Neighbor Notification Law, the city must opt into a state law requiring that commercial lawn pesticide applicators provide 48 hours’ advance notice to adjacent neighbors before spraying pesticides on lawns, trees, and shrubs.

“These bills put New York City at the forefront of the national effort to move pest control in a new direction, away from poisons and towards prevention,” says Laura Haight, senior environmental associate at the New York Public Interest Research Group, one of the organizations that spearheaded community-based campaigns for the laws.

Pesticides are extensively used in densely populated cities. Cockroaches, mice, and rats thrive in multifamily dwellings, where excessive moisture, structural cracks and crevices, abundant food sources, crowded apartments, and overstuffed closets provide nutrition and shelter for pests. In the New York City metropolitan area—which in the late 1990s accounted for more than a quarter of the total pesticide use in the state—these conditions are magnified by the sheer size of the urban center, where more than 8 million people live in 800 square kilometers.

“One of the most important potential effects from both laws may be the reduction of exposures to pesticides in schoolchildren,” says Claire Barnett, executive director of the Healthy Schools Network, an advocacy organization that helped push the laws through. It is expected that these laws could potentially reduce exposure to pesticides for over 1 million children in the city’s 1,500 public schools, as well as hundreds of thousands of other residents.

What made the NYC Pesticide Reduction Law feasible is that there are effective alternatives to pesticide use, says Barbara Brenner, principal investigator of an NIEHS-funded study at the Center for Children’s Environmental Health and Disease Prevention Research at Mount Sinai School of Medicine. Data published by Brenner’s group in the October 2003 *EHP* showed that reducing the breeding habitats for pests and using agents like boric acid that are nontoxic to humans effectively reduced cockroach infestation in an inner-city environment.

Says Brenner, “Cockroach, mouse, and rat infestation is a very real and serious problem in both indoor and outdoor environments throughout New York City. . . . However, traditional chemical pesticide spraying has not controlled the problem, bringing with it health risks and hazards of its own. Recognition of this dilemma by New York City government represents official recognition of both the problem and the need to now use proven least-toxic methods.”

City council member James Gennaro, who cosponsored both bills, says, “The active participation of community organizations and scientists were both vital to the success of this landmark legislation. . . . Frankly, I don’t believe this legislation would be law today without the involvement of these two essential groups.” He adds, “I firmly believe that this legislation will have tangible health benefits for large numbers of New York City residents.”

## Figures and Tables

**Figure:**